# Probabilistic Load Forecasting Optimization for Building Energy Models via Day Characterization

**DOI:** 10.3390/s21093299

**Published:** 2021-05-10

**Authors:** Eva Lucas Segarra, Germán Ramos Ruiz, Carlos Fernández Bandera

**Affiliations:** School of Architecture, University of Navarra, 31009 Pamplona, Spain; elucas@unav.es (E.L.S.); cfbandera@unav.es (C.F.B.)

**Keywords:** probabilistic load forecasting, day characterization, white-box models, building energy models, weather forecast, uncertainty analysis, monitoring, reliability, kernel density functions

## Abstract

Accurate load forecasting in buildings plays an important role for grid operators, demand response aggregators, building energy managers, owners, customers, etc. Probabilistic load forecasting (PLF) becomes essential to understand and manage the building’s energy-saving potential. This research explains a methodology to optimize the results of a PLF using a daily characterization of the load forecast. The load forecast provided by a calibrated white-box model and a real weather forecast was classified and hierarchically selected to perform a kernel density estimation (KDE) using only similar days from the database characterized quantitatively and qualitatively. A real case study is presented to show the methodology using an office building located in Pamplona, Spain. The building monitoring, both inside—thermal sensors—and outside—weather station—is key when implementing this PLF optimization technique. The results showed that thanks to this daily characterization, it is possible to optimize the accuracy of the probabilistic load forecasting, reaching values close to 100% in some cases. In addition, the methodology explained is scalable and can be used in the initial stages of its implementation, improving the values obtained daily as the database increases with the information of each new day.

## 1. Introduction

Smart cities, smart grids, and smart buildings [1,2,3], demand response and demand side management [4,5], renewable energy sources and their integration [6], nearly zero energy buildings [7], model predictive control [8], electric vehicles [9], etc., are perhaps some of the research areas that have received more emphasis in recent years. Global warming due to climate change, the scarcity of natural resources, and the growing global increase in energy demand are some of the reasons for this search for greater efficiency and smart energy use. In these fields, buildings play an important role since, for example, in the European Union (EU), they are responsible for 40% of energy consumption [10].

There are several approaches to reduce the energy consumption of buildings, which can be classified into two main groups: those based on “passive” strategies, understanding “passive” as those ones that do not require an active management of the building, as the improvement of building envelopes [11], the replacement of systems (HVAC, DHW, etc.) with more efficient ones [12], the use of renewable energies, etc.; and those based on “active” strategies that basically try to achieve an optimal management of the building as a whole [13,14].

Both approaches require reliable building energy models (BEMs) to test, verify, and quantify each measure and its improvements. However, characterizing a building in a simulation model is not an easy task [15]. In fact, there are different types of building energy models, depending on the methodology used to obtain them, which have their advantages and disadvantages [16]. They fall into three categories: black-box models, white-box models, and hybrid models. Black-box models are mathematical models based on data (data-driven models) that are generated from historical building information. They do not require physical definitions/settings or information about its internal loads or the characteristics of the environment, etc. They only need to be trained via the obtained data from the physical behavior of the actual building [17,18,19]. They are also often called surrogate models. Different approaches are used to obtain them, such as linear regressions, time series (ARMA and ARIMA) [20,21], machine learning [22,23], deep learning [24], etc., and their main advantage is that once trained, they allow simulations of their behavior in a short period of time; they are very fast models to simulate. However, any change in the building, its loads, or the environment implies a new training process, and if this change is significant, sometimes, they do not have enough data for the training to generate an accurate model. The second category, white-box models, are models based on physical principles. They take into account all the variables that define the building, both its formal characteristics and its loads and environment [25,26,27]. They do not require any training process, although they need a complete definition of all the aspects that affect the building’s energy demand and its subsequent adjustment to obtain reliable results (calibration). Their main disadvantage is that they require larger simulation times than black-box models; however, they are more flexible since building’s modifications do not require subsequent retraining [28,29]. They are the most recommended for applications whose objective is the prediction of the building’s energy behavior, since once the model is calibrated, it admits any variation of the boundary conditions, whether they are interior and/or exterior [30]. The software most widely used to perform white-box models are EnergyPlus and TRNSYS [31]. Finally, the third category corresponds to hybrid models. They are a combination of physics-based and data-driven models. They are used when it is easier to use data than to define some physical property of the model, and therefore, it is more convenient to combine both strategies [32,33].

There are different “active” strategies to optimize the use of buildings, such as demand response, demand side management, an optimum energy management system together with low voltage energy grids in different contexts, the appropriate use of the thermal inertia of buildings, the integration of distributed energy resources, etc. [5,34]. All of them have one key aspect in common: they all require accurate building energy demand/consumption forecasts.

This accuracy depends not only on the reliability of the model or on the indoor boundary conditions (internal loads, use schedules, etc.), but also on the weather, which is a key factor for obtaining an accurate load forecast [35,36]. That is why weather forecast has a great influence on the results obtained, with some weather parameters being more sensitive than others, such as outdoor temperature [37]. In spite of the importance and influence that weather forecast has on the simulation, the effect of its uncertainty has not been fully studied in the literature [38,39,40], and few research works have directly investigated its effect [35,37,41,42,43].

This is why a probabilistic approach is useful when analyzing the uncertainty of the energy demand/consumption obtained by using weather forecast, the probabilistic load forecast (PLF) [44]. Conventional load forecasting provides a specific value of the demand/consumption of the building, which is called point forecast. The problem is that this single value does not quantify the effect of model and weather uncertainties, so it is necessary to use a tool that describes the risk of the point forecast. The probabilistic load forecast allows defining these uncertainties and quantifying the risk [45].

Of the different techniques used to obtain a PLF, those based on statistical models are the most common, the non-parametric being the ones that offer greater flexibility since they adjust to both skewed or bimodal densities. As Van der Meer et al. [46] summarized, there are different strategies to build a non-parametric PLF such as quantile regression [47], quantile regression forest, Gaussian processes [48,49], Gaussian mixture models [50], bootstrapping, lower upper bound estimate, gradient boosting, kernel density estimation [51], k-nearest neighbor, etc. Among all of them, in this research, we used those based on kernel density estimation (KDE). A kernel is a standardized weighting function that assigns different weights to the observations. It depends on a parameter “*h*”, the bandwidth, which determines the amount of smoothing applied in the estimation [52]. Although obtaining the KDE function requires more computational time, the PLF results provide valid prediction intervals since the prediction interval coverage probability (PICP) values are in many cases above the prediction interval nominal confidence (PINC), which is 80% [53]. Furthermore, as Gonzales-Fuentes et al. [54] explained, “What KDE has in its favor is that it requires less samples to provide a good estimate”. This allows obtaining good PLFs in initial states, where the amount of data used to obtain the KDE function is low.

Research on probabilistic load forecasting is increasing in the literature; however, it is mainly focused on black-box models, which cannot show the link between the inputs and the forecast building loads. A previous research work from the authors Lucas Segarra et al. [53] aimed to fill this gap by providing a probabilistic load forecasting methodology that considers the weather prediction uncertainty using white-box models (BEMs), in particular calibrated BEMs using EnergyPlus [55], instead of black-box models. That methodology transforms the point load forecast provided by a BEM into a probabilistic load forecast using historical data based on outdoor and indoor conditions provided by building monitoring. The case study employed up to a total of 12 months of data (two years (two heating seasons)), and the results showed that the mean prediction interval width (MPIW) decreased as the amount of data used to perform the KDE function increased, maintaining good prediction interval coverage probabilities (PICPs).

This paper continued the research on PLF applied to white-box models or BEMs and went one step further and optimized the PLF values obtained by analyzing and classifying the data used to generate the KDE function. In this sense, it answers the question raised in that research on “how the seasonality of the weather data influences the results”.

To this end, a methodology was developed to characterize the days so that each KDE function is obtained using only information from days with similar characteristics, classified by different criteria, whether quantitative (amount of energy), qualitative (form of energy demand), or a mixture of both. Classifying each day is more logical than assuming seasonality in the data, since within each season, there will surely be days with different weather behaviors. Due to the purpose of the PLF being to obtain the demand/consumption of a specific day (short-term load forecasting) [56,57], this time period was used to obtain both the KDE function and its uncertainty map based on both quantitative and qualitative characteristics. Thus, when a load forecasting is made, it is first characterized in order to select the characterization criteria of the training days that will construct its uncertainty map. This enables obtaining more accurate PLFs, reaching in some cases, as will be seen in the case study, almost 100% accuracy.

To make the methodology scalable, a script that establishes a hierarchy in the cascade of all the proposed characterization criteria was developed. After analyzing and classifying the day under study, the script selects which criterion best meets each day. Obviously, at the beginning, not all the characterization criteria have enough information to obtain a valid KDE function, so the script selects the best among the existing ones and “feeds” each criterion with the data of the new day. As a result, this methodology can be used at the initial stages of each case study, since as the days go by, the data feedback allows classifying and improving the PLFs obtained.

The main contributions of the research are: the optimization of the probabilistic load forecasting using a characterization of the daily load forecast through the use of white-box models; and the continuous improvement of the results by feeding back the database as the days go by, allowing an early implementation.

This paper is organized as follows: Section 2 summarizes the calculation process of the PLF and of its uncertainty maps and explains the methodology to obtain an optimal PLF based on the days’ characterization. Section 3 focuses on the description of the case study for which the methodology was implemented, and Section 4 shows the results, including the evaluation of the methodology. Finally, Section 5 presents the discussion and conclusions of the study.

## 2. Methodology

This section explains first the probabilistic load forecast (PLF) approach using building energy models (BEMS) and accounting for weather forecast uncertainty and, then, the process to characterize the training days used to generate the optimized PLF and the uncertainty map.

### 2.1. Probabilistic Load Forecast Using BEMs

**Figure 1:**
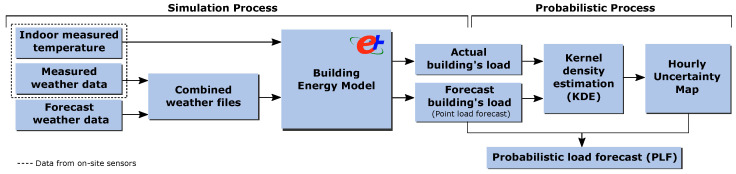
Components and steps of the probabilistic load forecasting procedure based on white-box models (building energy model (BEM)) [53].

This section explains the methodology to obtain probabilistic load forecasts taking into account the weather forecast uncertainty and using BEMs, which was previously published by the authors in [53]. The technique requires two procedures: the simulation process to determine the historical impact of the weather forecast data on the load provided by the BEM and the probabilistic processing of the simulation outputs. Figure 1 shows the overall schema of the probabilistic load forecasting methodology.

The first procedure is the simulation process. In order to reduce to a minimum the uncertainty in the load forecast due to building energy model accuracy, the proposed methodology employs calibrated building energy models (BEMs) based on EnergyPlus simulation software, which is an accurate representation of the behavior of the real building. Since the PLF procedure is based on the comparison of the load using observed and forecast weather data, it is required to explain how the forecast and observed data are implemented in the BEM and the simulation procedure, with special attention given to the weather files’ creation, the indoor temperature, and the simulation periods.

The weather files, in EPW (EnergyPlus weather file) format, are created respecting the thermal history of the building. The process of creating the weather file starts with the collection of daily forecast weather data from an external provider and the measured weather data from an on-site weather station installed in the building surroundings. Then, one weather file, called the combined weather file, is generated for each day with the measured weather information (historical data) and forecast data.

In order to correctly reflect the past thermal behavior in the BEM taking into account all loads of the building (HVAC system, people, lighting, electric equipment, etc.), the building’s indoor temperature measurements obtained from the sensors installed in the building are introduced into the model. The actual indoor temperature is introduced into the simulation model through an external file and configured as a dynamic set-point for the HVAC system. The simulation output is the energy demand required by the model to follow it. This way, the uncertainty in the load forecast due to internal gains and occupant behavior is avoided. In this sense, sensors installed in the building are fundamental devices since they connect the simulation model with the real world, increasing its accuracy by taking into account aspects that are difficult to virtualize [58].

**Figure 2:**
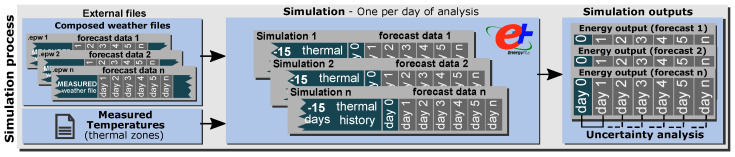
Simulation process methodology [53].

For each day of analysis, one simulation is performed, which is configured to run 15 days before the baseline day (Day 0) with the measured weather data to ensure that the thermal history of the building is captured. The loads on Day 0 and *n* days of the forecast are obtained as the outputs of each simulation. These results (ordered and classified) are subsequently used in the probabilistic process. The simulation process, the inputs (weather and measured temperature file), and the simulation period are explained in Figure 2.

**Figure 3:**
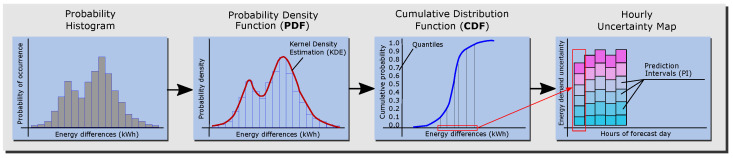
Process of the probabilistic load forecast [53].

The second procedure of the PLF technique employed is the probabilistic processing of the point forecasts, which are the simulation outputs obtained from the BEM. First, the distribution of the residuals, which are the energy load differences provided by the BEM when it is fed the observed and forecast weather data, is studied through a probabilistic histogram. Second, to obtain a smooth curve that represents the data, a probability density estimation is performed using the Gaussian kernel density estimate (KDE). The objective of this procedure is to obtain the expected probability that the load forecast error is below a certain value. The cumulative distribution function (CDF) or S-curve is employed to represent the probability that the variable (here, the load forecast error due to the weather forecast) will be less than or equal to a certain value. Finally, from the CDF plot of each forecast hour, prediction intervals (PIs) of load forecast errors are extracted and transformed into an hourly map of uncertainty. The schema in Figure 3 shows the complete process.

The resulting hourly map of the uncertainty of the load forecast due to weather forecast data shows an overview of the probability that the energy demand error is below a certain value for each hour. The map is constructed with the available hours ahead of the forecast time, and it is read as follows: for hour n, there is a% probability that the energy demand error is less than y kWh. This map can then be applied to the load forecast provided by the BEM by using the intervals of the energy demand error with their probabilities to the load forecast outputs of the model. The result is the load forecast with the probability error due to weather forecast data.

For a more detailed explanation of the PLF process, the previous paper from the authors can be consulted [53]. That study aimed at showing the methodology for providing a probabilistic load forecast using building energy models. The present study goes one step further and aims to provide an optimized PLF results by studying how to characterize the days used for the generation of the uncertainty map.

### 2.2. Optimal Probabilistic Load Forecast

This section shows how the PLF process explained in the previous section can be optimized by the correct selection of the training days that generate the PLF. The concept is that each day of study is individualized and has its own optimal training days in order to compare the future building’s energy behavior to similar days in the past. The steps to generate this optimal PLF for each day of study are four: (1) definition of the characterization criteria; (2) generation of the PLF for each one of the characterization criteria and for each day of the database; (3) analysis of the minimum training days needed for each characterization criteria so that the obtained PLF works better than the previous criteria; and (4) ordering the criteria to generate a hierarchical criteria application list. The following paragraphs give the explanations of the four steps.

#### 2.2.1. First Step: Definition of the Characterization Criteria

**Figure 4:**
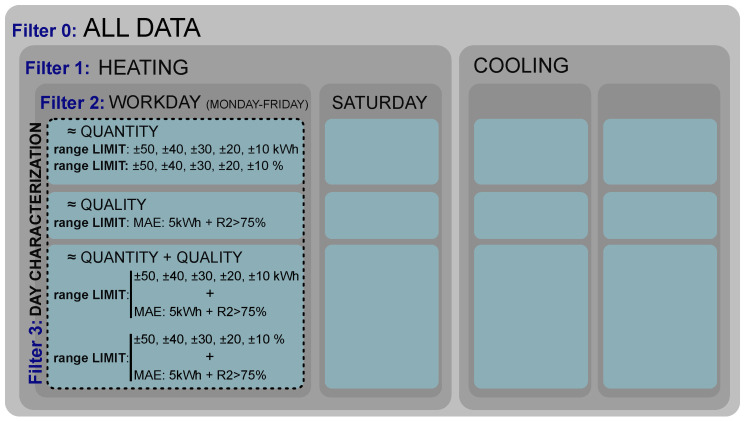
Hierarchy of filters proposed for the selection of the training days.

The first step is to define which are the characterization criteria when selecting the training days of the PLF. The key of the proposed methodology is that the selection of the data used for the generation of the uncertainty map and the PLF results is based on a daily unit and not on the seasonality of the data. The use of the seasonal or monthly criteria is discarded since it might happen that different days from different seasons cause a similar energy demand in the building regardless of the season or month in which they belong or that in a given month or season might be days with different behaviors that are negatively affecting the uncertainty map and PLF results. This methodology selects for each day of study similar days from the database attending to its energy demand. In order to characterize the days of the database and find the best set of training days for each day of study, four filters of selection criteria were established. These filters are ordered from highest to lowest filtrate grain, and one filter includes the previous one; in other words, they are cumulative. The schema of the proposed filters and the day characterization are shown in Figure 4.

Filter 0: baseline. All the database available was used for the uncertainty map calculation and PLF for each day of study. It should be highlighted that only days with energy demand requirements are included in this Filter 0. For example, if the HVAC system is turned off on Sundays, Sundays will not be part of the database.

Filter 1: filtered by type of energy demand. The database was divided in heating and cooling days. For example, when the day of study is a heating day, its uncertainty map is generated using all the heating days from the database.

Filter 2: filtered by the building’s use. It is usual that depending on the weekday, the building has a different use and HVAC schedule. For example, it is common that in an office building, working days and Saturdays have their own schedule. Therefore, this filter splits the database depending on the use of the building. In this research, working days and Saturdays were considered, but for other cases, this will depend on the specific use and schedule of the building of study.

Filter 3: filtered by similar energy demand. Days with a similar energy demand to the forecast energy demand of the day of study were selected for the creation of the uncertainty map. The energy demand was used to categorize the days instead other indicators as weather data since it reflects the energy effect that the combination of climatic parameters (outdoor temperature, humidity, solar radiation, etc.) generates in the building. Three different ways of identifying the similarity between the forecast energy demand of the day of study and the energy demand from the days of the data base were established:

1. Quantitative similarity: The days are similar in terms of the amount of energy demanded. This was analyzed using two different criteria: (1) by percentage with jumps of 5% with respect to energy demand (from 5% to 25%) and (2) by the amount of energy with jumps of 10 kWh (from 10 to 50 kWh). We selected 10 kWh because it matched with 10% of the mean daily energy demand of the building used in this study. For other cases with other mean daily energy demand, the jumps in kWh will be adjusted for the specific case.
2. Qualitative similarity: The days are similar in terms of the shape of the required energy demand curve. Two indexes were used for this characterization: mean absolute error (MAE) (Equation (1)), which measures the average magnitude of the error in the units of the variable of interest, and the coefficient of determination ($R^{2}$) (Equation (2)), which allows measuring the linear relationship of the two patterns [59]. A maximum limit for the MAE of 5 kWh and a minimum limit for $R^{2}$ of 75% were established.
  (1) $$MAE=\frac{1}{n}\sum_{i=1}^{n}\left|y_{i}-{\hat{y}}_{i}\right|,$$
  (2) $$R^{2}={\left(\frac{n\cdot \sum_{i=1}^{n}y_{i}\cdot \hat{y}-\sum_{i=1}^{n}y_{i}\cdot \sum_{i=1}^{n}\hat{y}}{\sqrt{\left(n\cdot \sum_{i=1}^{n}y_{i}^{2}-{\left(\sum_{i=1}^{n}y_{i}\right)}^{2}\right)\cdot \left(n\cdot \sum_{i=1}^{n}{\hat{y}}^{2}-{\left(\sum_{i=1}^{n}\hat{y}\right)}^{2}\right)}}\right)}^{2}$$
3. Combination of the quantitative and qualitative criteria: The days selected as training days fulfill at once the quantitative and qualitative criteria defined in each case.

#### 2.2.2. Second Step: PLF Calculation for Each Criterion

The second step is the PLF calculation for each day of the available database, which is performed for each one of the characterization criteria defined in the previous step. The PLF process follows the procedure explained in Section 2.1. For each day and criterion, three parameters are obtained: on the one hand, two indicators for the prediction interval assessment: the prediction interval coverage probability (PICP) and the mean prediction interval width (MPIW); and on the other hand, the number of training days with which the uncertainty map was generated, in other words the number of days that meet the criteria established in the previous step.

The PICP value measures the reliability of the predictions and shows the percentage of the real values that will are covered by the upper and lower bounds. The larger the PICP, the more likely that the real values are within the prediction interval. It can be defined as:

(3) $$PICP=\frac{1}{H}\sum_{i=1}^{H}C_{i},$$

in which *H* is the number of samples and $C_{i}$ is a Boolean variable defined as follows:

(4) $$C_{i}=\left\{\begin{array}{cc} 1, & y_{i}\in \left[L_{i},U_{i}\right]\\ 0, & y_{i}\notin \left[L_{i},U_{i}\right], \end{array}\right.$$

where $L_{i}$ and $U_{i}$ are the lower and upper PI bounds of target $y_{i}$, respectively. The PICP ranges between 0 and 100%. The prediction interval is considered valid if the PICP value is greater than the prediction interval nominal confidence (PINC=100(1−α)%), where α represents the probability of error.

A complementary metric is used to assess the prediction interval widths, the mean prediction interval width (MPIW), and it is defined as:

(5) $$MPIW=\frac{1}{H}\sum_{i=1}^{H}\left(U_{i}-L_{i}\right).$$

#### 2.2.3. Third Step: Definition of the Minimum Training Days

The third step defines what are the minimum training days required for each criterion since the ability of the PLF and the uncertainty map to foresee the error in the energy demand due to the weather forecast directly depends on the amount of training data used in the procedure. We analyzed the minimum number of training days required for each criterion to obtain improved results with respect to the results from the criterion of the previous filter.

The requirement that was established to consider that a criterion works better than the previous filter is that the average PICP of the days that meet the condition that have equal or more than x training days is greater than the average PICP of those same days for the criterion of the previous filter. The analysis was carried out for all available number of training days. At the moment in which, for a given minimum number of training days, the mean PICP of the criterion studied is higher than the mean PICP of the criterion of the previous filter, this criterion is considered to be better. It may happen that regardless of the number of days of training, the result of the average PICP does not exceed in any case that of the previous filter. In this case, it is considered that the criterion studied does not improve the previous filter, and it is discarded.

#### 2.2.4. Fourth Step: Ordering of the Characterization Criteria

After the analysis of the minimum training days, the criteria that do not generate improvements with respect to the criterion of the previous filter are discarded. Afterward, the criteria that do produce improvements are ordered according to the average PICP value. It is important to highlight that this PICP value is calculated using the days that meet the specific criterion. In this way, a hierarchy of criteria can be generated according to their ability to improve the PLF of the previous filter. The result is a list of criteria application ordered based on the ability to improve the PLF results.

Once the hierarchical list of criteria application is obtained based on the available data set, the process to use this methodology is applied as follows. First, the day of study is characterized by defining the type of energy demand for Filter 1 (heating or cooling) and the type of use of the building for Filter 2 (working day or Saturday). To define Filter 3, based on the similarity in the energy demand, the weather forecast is introduced in the BEM, and the required energy demand of the building is obtained. This energy demand is then compared with the forecast energy demand of the days from the database. This comparison allows counting how many days from the database meet the different criteria with respect the day of study; for example, how many days in the database have heating demand, are working days, and have and energy demand that is equal to or greater than 50 kWh with respect the forecast energy demand of the day of study. The last step is to apply the list of criteria application through a cascading process with conditional structure. The first criterion is selected, and it is analyzed if the minimum training days required are met. If the minimum training days required are fulfilled, this criterion is applied; if this is not fulfilled, the second criterion is selected to be analyzed, and so on, until one criterion meets the minimum training days. If the minimum training days requirement of any of the criteria for this Filter 3 is not met, the previous level of filtering (Filter 2) is applied. This procedure is explained below using a case study.

## 3. Description of the Case Study

**Figure 5:**
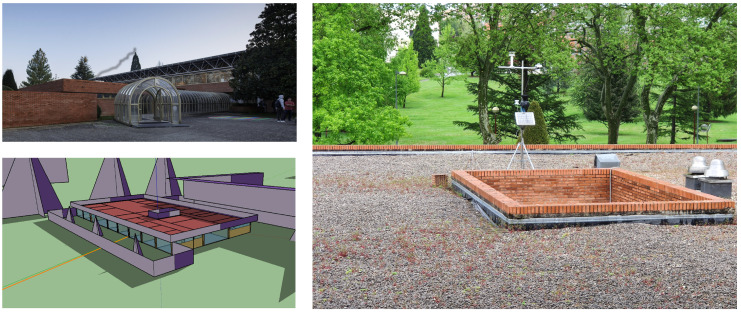
Building’s outdoor photograph (**top left**), simulation model image from OpenStudio [60] (**bottom left**), and the weather station located on the roof of the building (**right**).

In this section, the case study used to apply the proposed methodology is presented. The administrative building of the Architecture School at the Universidad de Navarra in Pamplona (Spain) was chosen to be the case study. This building, used for administration purposes and by postgraduate students of the School of Architecture, was built in 1974. It is a 760 square meter single-story building whose layout consists of a succession of staff offices, an administration area, an open work space, and classrooms. The building has a concrete structure. The walls are built of red brick fabric, and the windows have aluminum frames and air chambers. The building has a heating and cooling system. Figure 5 shows the building’s outdoor photograph, the weather station located on the building’s roof, and the simulation model. The building has an office schedule. On Saturdays, the building is used only in the mornings, and it is not used on Sundays.

The building energy model employed in the case study was developed using the EnergyPlus engine. In the load forecasting field, when using BEMs, it is important to take into account the three main sources of uncertainty: BEM accuracy, building use, and external conditions. This case study used a calibrated BEM, obtained using a calibration methodology explained in the authors’ previous papers [59,61,62,63,64]. Using a calibrated model allows minimizing the uncertainty due to BEM accuracy. Regarding the building’s use and its internal gains, no uncertainty was considered since the BEM used indoor temperatures measured in each thermal zone to take into account the indoor conditions, as explained in Section 2.1.

Regarding the external conditions, they were introduced into the model through the weather files, and both the observed and the forecast weather data were required to generated these files. The observed weather data were obtained from the weather station installed on the building, which provides measurements with hourly intervals for nine climate parameters: temperature, humidity, direct and diffuse irradiation, atmospheric pressure, rainfall, wind speed, and wind direction. Figure 5 shows the weather station location. Meteoblue [65] is the commercial service that is used to provide the forecast weather information. Meteoblue uses a multimodel/machine learning approach to calculate the forecast weather data using both its own meteorological models (nonhydrostatic meso-scale modeling) and third-party models for the simulations. More information about Meteoblue’s forecast weather data process is available on its web page [65]. In this study, the weather forecast data were obtained at 09:00 on each day.

The period in which all of the required data were available was from 13 June 2019 to 31 January 2020. The cooling system was connected from 13 June 2019 to 18 October 2019 and the heating system from 19 October 2019 to 31 January 2020.

In the following sections, the methodology is illustrated showing how the four steps explained in Section 2.2 were applied in this case study. Then, we evaluate the ability of the proposed methodology to get optimized PLF results by characterizing the training days applying the methodology to the last 15 working days of the data set available (validation period).

## 4. Results

In this section, the results of applying the proposed methodology to get optimized PLF results by characterizing the training days are presented. Due to the paper’s extent, the results of the case study were focused on days when the heating system was turned on and were classified as working days. The four steps explained in Section 2 are presented using the data from the case study, and the methodology was validated showing the PLF results with the four levels of filtering using the last 15 working days of January 2020 (validation period).

First Step: Definition of the characterization criteria

First, the characterization criteria were established. The following Table 1 shows the four characterization filters of the training days for the calculation of the PLFs. Within the Filter 3, similar energy demand, it shows as well the three ways of identifying the similarity between the forecast energy demand of the day of study and the energy demand from the days of the data set.

Second Step: PLF calculation for each criterion

**Figure 6:**
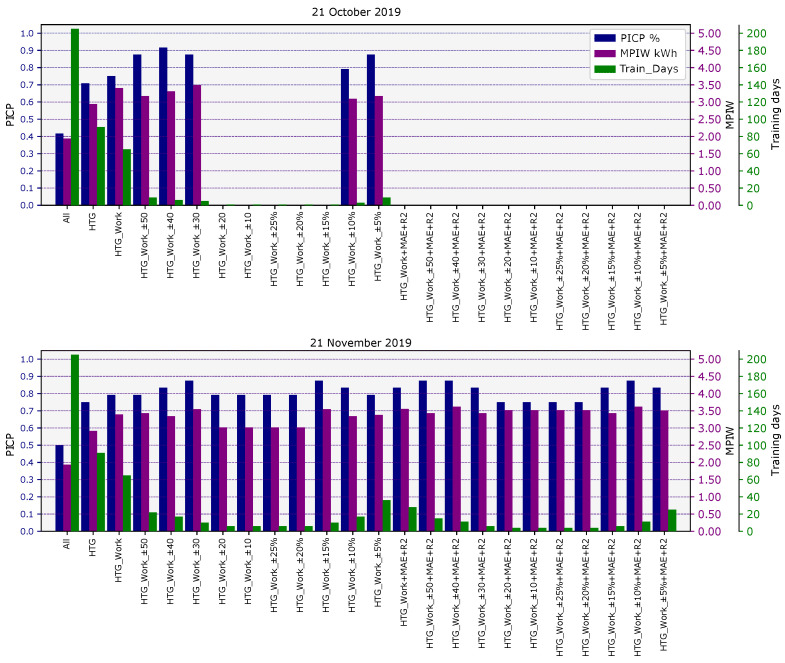
PICP, MPIW, and training days for the different filters and days’ characterization. **Top**: 21 October 2019. **Bottom**: 21 November 2019.

For each day of the available database, the PLF calculation was performed for each characterization criteria, from Filter 0 to Filter 3. For each case, we obtained the PICP and MPIW values and the number of training days, that is the number of days that met the criteria and with which the PLF map was generated. The following graph Figure 6 shows the results for two sample days (21 October 2019 and 21 November 2019). The graph shows the PICP, MPIW, and training days used in the PLF for all criteria. In the case of 21 November 2019, all criteria had data to generate the uncertainty map; in other words, there was at least one day in the database that met the requirements of each criterion. However, in the example of 21 October 2019, many criteria did not have training days available that met the requirement in each case, especially for qualitative similarities. The graphs also show how the characterization of the days improved the results gradually. For example, on 21 October 2019, the PICP value for All (Filter 0) started at 41% (below the confidence level) and it grows to 70% for HTG (Filter 1), to 75% for HTG-Work (Filter 2) and from 80% to 90% for the criteria from Filter 3, which is above the confidence level (80%).

The following Table 2 shows the results for the 66 heating working days of the data set. The first column indicates the filter to which the criterion belongs. The second indicates the selection criteria for training days. The third column shows the number of days with respect to the 66 days in which there was training that met the criteria. For example, in the case of HTG-Work±20%+MAE+R2, only 38 days of the 66 had at least one day of training. The fourth column “training days” shows the days (or ranges of days) with which the uncertainty maps were constructed for each criterion. The fifth column shows the mean PICP for the days in which the uncertainty map was available, and the sixth shows the mean MPIW for those days.

Regarding the results shown in this table, in the first three filters, a clear improvement in the mean PICP was appreciated as the filter gets smaller, going from a mean PICP of 55.9% for the All criterion, where the training days included all the days from the database, at a mean PICP of 83% when only the HTG and working days were used to generate the maps (HTG-Work). In Filter 3, when similar energy demand filters were applied in the training days’ selection criteria, not every day from the database was a possible training day that met these criteria; therefore, most filters had less than 66 days. On the other hand, the range of training days for each criterion was very wide, for example from one to 36 days in the case of HTG-Work±5%. That is, within the same criteria, there were days that had very few training days, and other days had many training days since it depended on the forecast load provided by the BEM and the available data from the past. It should be noted that the table shows the mean PICP, but the PICP results for each day were very different depending on the number of training days available. For example, in the case of HTG-Work±5%, although the mean PICP was 82.5%, there were some days that achieved PICP values of 100% when there were enough available training days. Therefore, the next step was to analyze from how many days of training the result of applying Filter 3 improved the indices of the previous filter (Filter 2, HTG-Work), that is define the minimum days of training for each criterion to be effective.

Third Step: Definition of the minimum training days

The third step analyzes the minimum training days for each criterion. The following Table 3 shows for each criterion from Filter 3 (filtered by similar energy demand) the minimum training days required by each criterion to improve the PICP value from the previous filter (Filter 2, HTG-Work) and the values of the mean PICP for both Filters 2 and 3. The table shows that six criteria from Filter 3 did not achieve an improvement in the mean PICP values. In Appendix A, we present Table A1, Table A2 and Table A3 showing how the minimum training days were selected.

Fourth Step: Ordering of the characterization criteria

The aim of this step is to define the order of application of the characterization criteria. Once the minimum training days necessary for each criterion to improve the results of the previous filter have been established, the criteria that do not imply an improvement in these results are discarded. In this case study, six criteria of the 21 initially proposed did not improve the PICP results of Filter 2 and, consequently, were discarded. These six criteria are marked in the previous Table 3 with a hyphen. The 15 criteria that succeeded in improving the PICP results of the previous Filter 2 were ordered according to the mean PICP obtained. Table 4 shows these criteria ordered by the PICP (from 94.4% of the first criteria to 76.2% to the fifteenth) and the minimum required training days to be applied.

The table above is the hierarchical list of criteria application ordered by their ability to improve the PLF results. The following section presents how this was used.

### Validation of the Methodology

This section presents how the methodology was applied, showing the results obtained for the last 15 heating working days of the available data set. These were Days 10, 14–17, 20–24, and 27–31 of January, 2020. The following Table 5 shows an example, using the last day of the test days (31 January 2020), of how the criterion from Filter 3 (filtered by similar energy demand) was selected. The table shows the order of application of the criteria as calculated in the previous section and the minimum training days necessary to obtain an improvement with respect to the criterion of the previous filter (Filter 2, HTG-Work). The table also indicates the number of training days for this specific day of study (in this example, 31 January 2020) and if the minimum days were met in each criterion. The table is given in descending order, and the first criterion in which the minimum training days were met is the one to be selected, in this case HTG-Work±5%. In the event that the minimum days of training were not met for any criterion, the criterion of the previous filter (Filter 2, HTG-Work) would be applied.

The results for the testing days (the last 15 working days of January 2020) are shown in the following Table 6. It compares the results of the PICP and MPIW values for each one of the days and for each one of the filters (from Filter 0 to Filter 3), as well as the mean of all the days. It is observed how the PICP increases as the characterization of the day grows. The MPIW also increases, but in a measured way. Regarding the jump from Filter 2 to Filter 3, Filter 2 being the filter employed in the previous paper from the authors, it should be noted that the mean PICP of the days studied grew from 78.6% to 82.2%, that is by using the energy demand similarity filter, the mean PICP exceeded the confidence level. Analyzing the results for each day, it was observed that of the 15 days, fourteen days had a PICP equal to or greater than Filter 2, HTG-Work PICP, and of them, eight increased the PICP results. Only one day (15 January 2020) provided a lower PICP when applying Filter 3 (83.3%) compared to Filter 2 (87.5%), but it should be noted that the result was still above the confidence level (80%).

**Figure 7:**
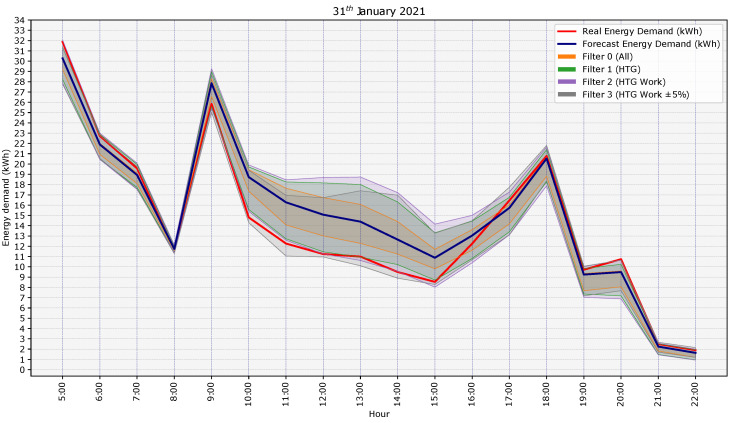
Graphical results of the PLF for the four filters for the last day of the test period, 31 January 2020.

In order to show graphically how the prediction of the building’s energy demand improved as the characterization of the day was adjusted using similar training days to the day of study, a graph with the results for a day of study is shown in Figure 7. Specifically, we show the last day of the test period, 31 January 2020. The graph shows the real energy demand (red line), the forecast energy demand provided by the model using the weather forecast (blue line), and the probabilistic load forecast for each filter. It was observed how as the characterization of the days was adjusted and the filter more accurate (from Filter 0 in orange shadow to Filter 3 in grey shadow), the PLF adapted better to the real energy demand curve, since the shadow of the prediction intervals better covered the real energy demand. Applying the criterion of Filter 3, where the similarity of the energy demand was applied, means significantly improving the PLF. For the day presented in the graph, the PICP values increased from 12.5% for Filter 0 (All) to 87.5% for Filter 3 (HTG-Work±5%) (see the results in Table 6). The better adjustment of the PLF to the real energy demand can be seen graphically especially between Hours 10 and 12, when the real energy demand only fell within the PLF range obtained with the criterion of Filter 3.

## 5. Conclusions

In the current energy context, load forecasting is necessary to optimize the use of energy in buildings. Forecasting future energy demand necessarily requires dealing with the effect of uncertainties. One of the most important uncertainties and that affect the load forecasting results more is weather forecast. Traditional load forecasts based on point predictions are not able to reflect properly its effect on the energy forecasts. However, probabilistic load forecast allows taking into account the uncertainties in the energy load forecast. Previous research from the authors presented a methodology that provides the probabilistic load forecast while accounting for the inherent uncertainty in forecast weather data using a white-box model (BEM). The result is an hourly map of the uncertainty of the load forecast, which allows the point load forecasting provided by the BEM to be converted into a probabilistic load forecast. The methodology fixes the other uncertainties such as building model accuracy and user behavior in order to focus the research on the weather forecast uncertainty. This study aimed to go one step further and get optimized PLF results by providing a methodology to select the best amount of data for the training days that generates the map of uncertainty. The methodology was based on each day of study being individualized and it being provided with its own optimal training days in order to compare the future building’s energy behavior to similar days in the past.

A case study in a real office building in Pamplona, Spain, was presented. Eight months of data from the building’s monitoring, weather station, and weather forecast were available for the study with the cooling system connected from June 2019 to October 2019 and the heating system from October 2019 to January 2020. The methodology establishes four levels of filtering: Filter 0, baseline with all the data available; Filter 1, filtered by type of energy demand; Filter 2, filtered by the type of use of the building; and Filter 3, filtered by similar energy demand. The similarity of the energy demand from Filter 3 is identified by three different ways: quantitative similarity, qualitative similarity, and a combination of both.

As an example of applying the methodology in a real case scenario, the paper showed how the methodology was developed, focusing on working days that have the heating system, and it is validated by applying it to the last 15 working days of the data available (last 15 working days of January 2020). The PICP and MPIW indicators were used to compared the results for each filter. The results showed how the PICP value increased as more accurate filters when selecting the training days were applied. Analyzing Filter 3 application and comparing it to the previous Filter 2, which was the filter employed in the previous paper from the authors, it was seen how the mean PICP grew from 78.6% to 82.2%, which exceeded the confidence level when the energy demand similarity filter was applied, and PICP values up to 95.8% were obtained for some testing days. The use of the energy demand characterization criteria of Filter 3 provided that 14 days of the 15 days of study achieved a PICP value equal to or greater than the previous filter criterion, and eight of them increased this value.

The results showed how important the selection of the training days for the generation of the uncertainty map and the PLF calculation is. The paper showed as well how individualizing the training days and selecting them using the days characterization proposed by this methodology considerably improved the PICP results, which means that this methodology reached better probabilistic load forecasts. In conclusion, the application of this methodology allows obtaining an optimized prediction of the near-future energy demand of the building taking into account the weather forecast uncertainty, one of the most important sources of uncertainty in the building load prediction field. It is a useful tool for any application in which the future load forecast is required.

One important characteristic of this methodology is that it is constantly fed back the newly measured and gathered data from the building, weather station, and weather forecast provider. As the available data increase, the methodology gains robustness since the procedure is based on the energy characterization of the training days that configured the uncertainty map and the PLF, and the more data, the more possible training days for each criterion there are. The methodology can be easily automatized to directly incorporate the new data to redo the PLF calculations for each criterion. 

## Figures and Tables

**Table 1:** Characterization filters of the training days for the calculation of the PLFs.

Filter 0	Baseline	All			
**Filter 1**	**Type Energy** **Demand**	**HTG**		**CLG**	
**Filter 2**	**Type Use** **Building**	**HTG_Work**	**HTG_Sat**	**CLG_Work**	**CLG_Sat**
**Filter 3**	**Similar Energy** **Demand**				
	Quantitative	HTG_Work_±50	HTG_Sat_±50	CLG_Work_±50	CLG_Sat_±50
	similarity	HTG_Work_±40	HTG_Sat_±40	CLG_Work_±40	CLG_Sat_±40
		HTG_Work_±30	HTG_Sat_±30	CLG_Work_±30	CLG_Sat_±30
		HTG_Work_±20	HTG_Sat_±20	CLG_Work_±20	CLG_Sat_±20
		HTG_Work_±10	HTG_Sat_±10	CLG_Work_±10	CLG_Sat_±10
		HTG_Work_±25%	HTG_Sat_±25%	CLG_Work_±25%	CLG_Sat_±25%
		HTG_Work_±20%	HTG_Sat_±20%	CLG_Work_±20%	CLG_Sat_±20%
		HTG_Work_±15%	HTG_Sat_±15%	CLG_Work_±15%	CLG_Sat_±15%
		HTG_Work_±10%	HTG_Sat_±10%	CLG_Work_±10%	CLG_Sat_±10%
		HTG_Work_±5%	HTG_Sat_±5%	CLG_Work_±5%	CLG_Sat_±5%
	Qualitative similarity	HTG_Work+MAE+R2	HTG_Sat+MAE+R2	CLG_Work+MAE+R2	CLG_Sat+MAE+R2
	**Combination**	HTG_Work_±50+MAE+R2	HTG_Sat_±50+MAE+R2	CLG_Work_±50+MAE+R2	CLG_Sat_±50+MAE+R2
		HTG_Work_±40+MAE+R2	HTG_Sat_±40+MAE+R2	CLG_Work_±40+MAE+R2	CLG_Sat_±40+MAE+R2
		HTG_Work_±30+MAE+R2	HTG_Sat_±30+MAE+R2	CLG_Work_±30+MAE+R2	CLG_Sat_±30+MAE+R2
		HTG_Work_±20+MAE+R2	HTG_Sat_±20+MAE+R2	CLG_Work_±20+MAE+R2	CLG_Sat_±20+MAE+R2
		HTG_Work_±10+MAE+R2	HTG_Sat_±10+MAE+R2	CLG_Work_±10+MAE+R2	CLG_Sat_±10+MAE+R2
		HTG_Work_±25%+MAE+R2	HTG_Sat_±25%+MAE+R2	CLG_Work_±25%+MAE+R2	CLG_Sat_±25%+MAE+R2
		HTG_Work_±20%+MAE+R2	HTG_Sat_±20%+MAE+R2	CLG_Work_±20%+MAE+R2	CLG_Sat_±20%+MAE+R2
		HTG_Work_±15%+MAE+R2	HTG_Sat_±15%+MAE+R2	CLG_Work_±15%+MAE+R2	CLG_Sat_±15%+MAE+R2
		HTG_Work_±10%+MAE+R2	HTG_Sat_±10%+MAE+R2	CLG_Work_±10%+MAE+R2	CLG_Sat_±10%+MAE+R2
		HTG_Work_±5%+MAE+R2	HTG_Sat_±5%+MAE+R2	CLG_Work_±5%+MAE+R2	CLG_Sat_±5%+MAE+R2

**Table 2:** PLF calculation results for heating weekdays.

#	Filter	No. of Days Met Filter	Training Days PLF	Mean PICP	Mean MPIW
0	All	66	205	55.9%	1.94
1	HTG	66	91	79.1%	2.93
2	HTG_Work	66	65	83.0%	3.4
3	HTG_Work_±50	66	2–25	80.7%	3.44
	HTG_Work_±40	66	2–21	80.0%	3.47
	HTG_Work_±30	65	2–15	79.6%	3.51
	HTG_Work_±20	60	1–11	76.3%	3.26
	HTG_Work_±10	52	1–8	71.0%	2.75
	HTG_Work_±25%	56	1–11	75.4%	3.25
	HTG_Work_±20%	58	1–12	76.7%	3.35
	HTG_Work_±15%	58	1–15	79.9%	3.36
	HTG_Work_±10%	62	1–21	79.9%	3.42
	HTG_Work_±5%	64	1–36	82.3%	3.34
	HTG_Work+MAE+R2	56	1–31	78.6%	3.28
	HTG_Work_±50+MAE+R2	51	1–17	75.0%	2.88
	HTG_Work_±40+MAE+R2	48	1–15	75.7%	2.9
	HTG_Work_±30+MAE+R2	45	1–11	77.7%	3.02
	HTG_Work_±20+MAE+R2	40	1–9	71.3%	2.71
	HTG_Work_±10+MAE+R2	28	1–5	65.5%	2.39
	HTG_Work_±25%+MAE+R2	36	1–8	67.1%	2.83
	HTG_Work_±20%+MAE+R2	38	1–9	73.4%	2.99
	HTG_Work_±15%+MAE+R2	42	1–11	78.7%	2.96
	HTG_Work_±10%+MAE+R2	43	1–15	77.7%	3.02
	HTG_Work_±5%+MAE+R2	51	1–26	78.2%	3.04

**Table 3:** Minimum training days required by each criterion.

Filter	Minimum Training Days	Mean PICP Filter 2 (HTG_Work)	Mean PICP Filter 3
HTG_Work_±50	7	82.2%	82.4%
HTG_Work_±40	5	81.5%	81.8%
HTG_Work_±30	8	82.3%	82.7%
HTG_Work_±20	-	-	-
HTG_Work_±10	-	-	-
HTG_Work_±25%	-	-	-
HTG_Work_±20%	9	84.4%	85.6%
HTG_Work_±15%	4	81.4%	82.5%
HTG_Work_±10%	7	81.4%	81.5%
HTG_Work_±5%	3	82.1%	83.4%
HTG_Work+MAE+R2	4	82.7%	83%
HTG_Work_±50+MAE+R2	15	82.4%	82.8%
HTG_Work_±40+MAE+R2	-	-	-
HTG_Work_±30+MAE+R2	9	81.9%	83.3%
HTG_Work_±20+MAE+R2	7	75.8%	76.2%
HTG_Work_±10+MAE+R2	-	-	-
HTG_Work_±25%+MAE+R2	-	-	-
HTG_Work_±20%+MAE+R2	8	82.6%	83.8%
HTG_Work_±15%+MAE+R2	6	82.6%	82.8%
HTG_Work_±10%+MAE+R2	14	93.1%	94.4%
HTG_Work_±5%+MAE+R2	3	81.2%	81.9%

**Table 4:** Ordering of the characterization criteria for the heating working days employed in the study.

Order	Filter 3	Mean PICP	Min Training Days
1	HTG_Work_±10%+MAE+R2	94.4%	14
2	HTG_Work_±20%	85.6%	9
3	HTG_Work_±20%+MAE+R2	83.8%	8
4	HTG_Work_±5%	83.4%	3
5	HTG_Work_±30+MAE+R2	83.3%	9
6	HTG_Work+MAE+R2	82.8%	4
7	HTG_Work_±15%+MAE+R2	82.8%	6
8	HTG_Work_±50+MAE+R2	82.8%	15
9	HTG_Work_±30	82.7%	8
10	HTG_Work_±15%	82.5%	4
11	HTG_Work_±50	82.4%	7
12	HTG_Work_±5%+MAE+R2	81.9%	3
13	HTG_Work_±40	81.8%	5
14	HTG_Work_±10%	81.5%	7
15	HTG_Work_±20+MAE+R2	76.2%	7

**Table 5:** Selection of the criterion from Filter 3 (filtered by similar energy demand).

Order	Filter 3	Min Training Days	Training Days 31 January 2020	Fulfills Training Days?
1	HTG_Work_±10%+MAE+R2	14	1	No
2	HTG_Work_±20%	9	2	No
3	HTG_Work_±20%+MAE+R2	8	1	No
**4**	**HTG_Work_±5%**	**3**	**13**	**Yes**
5	HTG_Work_±30+MAE+R2	9	3	No
6	HTG_Work+MAE+R2	4	18	Yes
7	HTG_Work_±15%+MAE+R2	6	2	No
8	HTG_Work_±50+MAE+R2	15	5	No
9	HTG_Work_±30	8	7	No
10	HTG_Work_±15%	4	3	No
11	HTG_Work_±50	7	11	Yes
12	HTG_Work_±5%+MAE+R2	3	6	Yes
13	HTG_Work_±40	5	8	Yes
14	HTG_Work_±10%	7	5	No
15	HTG_Work_±20+MAE+R2	7	2	No

**Table 6:** PICP and MPIW results for the validation period for the four filters.

Filter		Filter 0			Filter 1			Filter 2			Filter 3		
		All			HTG			HTG_Work			HTG_Work_()		
Date		PICP	MPIW		PICP	MPIW		PICP	MPIW		PICP	MPIW	Filter Selected
10/01/2020		54.2%	1.94		87.5%	2.93		91.7%	3.42		91.7%	4.15	_HTG_Work_±20%
14/01/2020		50.0%	1.94		75.0%	2.93		83.3%	3.39		95.8%	3.96	_HTG_Work_±5%
15/01/2020		58.3%	1.95		87.5%	2.92		87.5%	3.41		83.3%	3.59	_HTG_Work_±20%
16/01/2020		29.2%	1.94		66.7%	2.92		70.8%	3.39		70.8%	3.43	_HTG_Work_±5%
17/01/2020		58.3%	1.95		87.5%	2.93		91.7%	3.40		95.8%	3.44	_HTG_Work_±5%
20/01/2020		62.5%	1.94		70.8%	2.91		75.0%	3.36		75.0%	3.81	_HTG_Work_±5%
21/01/2020		41.7%	1.93		62.5%	2.90		66.7%	3.35		75.0%	3.94	_HTG_Work_±5%
22/01/2020		54.2%	1.93		66.7%	2.91		66.7%	3.40		66.7%	3.68	_HTG_Work_±5%
23/01/2020		50.0%	1.94		66.7%	2.92		70.8%	3.38		83.3%	3.61	_HTG_Work_±20%
24/01/2020		54.2%	1.94		87.5%	2.93		87.5%	3.41		91.7%	3.53	_HTG_Work_±5%
27/01/2020		79.2%	1.95		95.8%	2.94		95.8%	3.43		95.8%	3.98	_HTG_Work_±5%
28/01/2020		54.2%	1.94		58.3%	2.92		62.5%	3.40		66.7%	3.45	_HTG_Work_±5%
29/01/2020		45.8%	1.93		66.7%	2.93		75.0%	3.40		75.0%	3.41	_HTG_Work_±5%
30/01/2020		25.0%	1.93		70.8%	2.92		75.0%	3.37		79.2%	3.48	_HTG_Work_±5%
31/01/2020		12.5%	1.93		70.8%	2.90		79.2%	3.39		87.5%	3.23	_HTG_Work_±5%
Mean		48.6%	1.94		74.7%	2.92		78.6%	3.39		82.2%	3.65	

## Abbreviations

The following abbreviations are used in this manuscript:

### Abbreviations

ARIMA	Autoregressive integrated moving average
ARMA	Autoregressive moving average
BEM	Building energy model
CDF	Cumulative distribution function
CLG	Cooling
DHW	Domestic hot water
EPW	EnergyPlus weather file
HTG	Heating
HVAC	Heating, ventilation, and air conditioning
KDE	Kernel density estimation
MAE	Mean absolute error
MPIW	Mean prediction interval width
PDF	Probability density function
PI	Prediction intervals
PICP	Prediction interval coverage probability
PINC	Prediction interval nominal confidence
PLF	Probabilistic load forecast
Sat	Saturday
$R^{2}$	Coefficient of determination

## Appendix A

The following Table A1, Table A2 and Table A3 show how the minimum training days of the case study were defined. The tables present for each criterion from Filter 3 (filtered by similar energy demand) the comparison between the mean PICP applying the energy demand similarity filter studied in each case and the mean PICP of the criterion of the previous filter (HTG-Work), for all minimum training days. It should be noted that, in order to allow the comparison between criteria, the mean PICP in both cases is calculated for the days that have the minimum number of training days analyzed in each case available. The table allows quickly seeing for which minimum number of training days the results are improved when applying the energy demand similarity criterion from Filter 3. These minimum training days are highlighted in the table for each criterion with a box. It is remarkable that for some criteria, it is not possible to improve the HTG-Work criteria.

**Table A1:** Definition of the minimum number of training days required for each criterion within Filter 3. The highlighted cells show when the mean PICP of each criterion of Filter 3 improves the mean PICP of Filter 2.

Min Training Days		HTG_Work_±50			HTG_Work_±40			HTG_Work_±30			HTG_Work_±20			HTG_Work_±10			HTG_Work_±25%			HTG_Work_±20%	
		Mean PICP			Mean PICP			Mean PICP			Mean PICP			Mean PICP			Mean PICP			Mean PICP	
		Filter 2	Filter 3		Filter 2	Filter 3		Filter 2	Filter 3		Filter 2	Filter 3		Filter 2	Filter 3		Filter 2	Filter 3		Filter 2	Filter 3
1		82.97%	80.69%		82.97%	80.01%		82.71%	79.60%		82.58%	76.29%		83.37%	70.99%		81.86%	75.39%		81.62%	76.68%
2		82.97%	80.69%		82.97%	80.01%		82.71%	79.60%		82.64%	77.10%		83.37%	71.60%		81.91%	76.24%		81.62%	76.68%
3		82.57%	81.85%		82.29%	81.97%		82.14%	81.03%		82.11%	78.35%		82.84%	76.08%		81.84%	79.73%		81.99%	79.47%
4		82.42%	81.90%		82.14%	82.03%		82.29%	80.99%		81.32%	79.56%		82.98%	77.53%		81.76%	79.13%		81.84%	80.34%
5		82.14%	82.02%		81.54%	81.85%		82.06%	81.27%		82.22%	80.39%		84.72%	82.99%		83.06%	79.93%		81.51%	80.04%
6		81.98%	81.79%		81.67%	81.04%		81.86%	81.04%		82.41%	79.89%		85.83%	85.42%		83.58%	79.91%		83.58%	82.06%
7		82.22%	82.37%		81.86%	81.34%		82.12%	81.44%		83.65%	80.83%		77.78%	75.00%		83.83%	79.39%		83.39%	82.16%
8		81.91%	82.30%		81.86%	81.34%		82.33%	82.65%		82.47%	78.86%		83.33%	83.33%		82.88%	79.09%		83.58%	82.81%
9		81.81%	82.44%		81.45%	80.31%		82.96%	82.27%		80.16%	78.65%					82.87%	79.54%		84.39%	85.56%
10		81.75%	82.40%		81.52%	80.33%		82.96%	82.27%		82.58%	79.92%					89.17%	83.17%		84.02%	84.85%
11		82.16%	82.70%		81.82%	80.48%		82.72%	82.23%		85.42%	81.25%					91.67%	83.33%		82.42%	83.33%
12		81.93%	82.68%		82.68%	81.31%		80.33%	79.67%											75.00%	80.56%
13		82.01%	82.75%		82.66%	81.16%		77.92%	76.85%												
14		81.67%	82.16%		82.33%	81.00%		75.28%	75.69%												
15		82.33%	82.56%		82.59%	80.70%		68.75%	64.58%												
16		82.33%	82.56%		82.73%	80.81%															
17		83.16%	83.08%		82.66%	82.46%															
18		83.16%	83.08%		85.53%	84.39%															
19		83.74%	83.52%		84.72%	82.64%															
20		82.04%	82.04%		87.50%	79.17%															
21		83.86%	83.86%		87.50%	79.17%															
22		86.46%	86.46%																		
23		87.50%	86.90%																		
24		91.67%	89.58%																		
25		91.67%	91.67%																		

**Table A2:** Definition of the minimum number of training days required for each criterion within Filter 3. The highlighted cells show when the mean PICP of each criterion of Filter 3 improves the mean PICP of Filter 2.

Min Training Days		HTG_Work_±15%			HTG_Work_±10%			HTG_Work_±5%			HTG_Work			HTG_Work_±50			HTG_Work_±40			HTG_Work_±30	
											+MAE+R2			+MAE+R2			+MAE+R2			+MAE+R2	
		Mean PICP			Mean PICP			Mean PICP			Mean PICP			Mean PICP			Mean PICP			Mean PICP	
		Filter 2	Filter 3		Filter 2	Filter 3		Filter 2	Filter 3		Filter 2	Filter 3		Filter 2	Filter 3		Filter 2	Filter 3		Filter 2	Filter 3
1		81.62%	79.94%		82.14%	79.85%		82.50%	82.32%		83.27%	78.59%		82.53%	75.03%		82.93%	75.75%		83.12%	77.67%
2		81.62%	79.94%		82.14%	79.85%		82.50%	82.32%		83.27%	78.59%		82.53%	75.03%		82.93%	75.75%		82.54%	77.54%
3		81.30%	79.97%		81.98%	80.61%		82.14%	83.36%		82.52%	81.77%		82.03%	79.50%		81.90%	81.15%		81.90%	80.21%
4		81.36%	82.45%		81.30%	81.08%		82.14%	83.36%		82.74%	83.00%		81.90%	81.35%		82.08%	81.54%		82.30%	81.86%
5		81.70%	81.83%		81.53%	81.45%		81.84%	83.50%		82.35%	82.91%		81.90%	81.35%		82.69%	80.79%		83.41%	82.58%
6		81.37%	81.68%		81.52%	81.51%		81.37%	83.15%		81.72%	82.69%		82.16%	81.35%		82.69%	80.79%		83.14%	82.36%
7		82.35%	82.59%		81.36%	81.52%		81.59%	83.80%		81.62%	82.85%		82.30%	80.83%		82.27%	80.51%		83.67%	82.88%
8		82.23%	82.77%		81.70%	81.12%		81.59%	83.80%		81.62%	82.85%		82.24%	81.20%		82.53%	79.88%		81.90%	80.69%
9		81.71%	82.50%		81.84%	80.99%		81.59%	83.80%		81.15%	82.52%		83.28%	81.01%		83.60%	80.13%		81.87%	83.26%
10		82.50%	82.89%		81.72%	81.02%		81.53%	83.82%		81.97%	83.05%		83.28%	81.01%		83.45%	79.92%		77.38%	79.64%
11		82.15%	82.24%		82.40%	81.16%		81.53%	83.82%		81.97%	83.05%		83.73%	81.45%		83.07%	79.61%		75.00%	70.83%
12		82.24%	83.59%		81.65%	80.45%		81.53%	83.82%		81.97%	83.05%		84.65%	81.91%		84.58%	78.33%			
13		78.33%	79.55%		81.81%	80.58%		81.53%	83.82%		82.01%	83.38%		85.48%	83.29%		79.17%	72.50%			
14		77.31%	79.17%		81.24%	79.68%		81.31%	83.60%		82.01%	83.38%		83.83%	82.44%		87.50%	87.50%			
15		83.33%	79.17%		82.75%	81.26%		81.36%	83.61%		82.01%	83.38%		82.41%	82.78%		87.50%	87.50%			
16					82.81%	81.45%		81.36%	83.61%		84.58%	85.86%		80.56%	83.06%						
17					83.26%	82.77%		81.84%	83.54%		84.81%	86.44%		89.58%	85.42%						
18					84.52%	85.12%		81.84%	83.54%		84.66%	85.86%									
19					87.04%	87.04%		81.84%	83.54%		84.87%	85.80%									
20					93.75%	87.50%		81.84%	83.54%		84.83%	86.01%									
21					87.50%	79.17%		81.84%	83.54%		84.83%	86.01%									
22											84.17%	86.12%									
23											83.45%	85.49%									
24											84.25%	86.39%									
25											84.25%	86.39%									
26											84.78%	87.59%									
27											84.53%	87.08%									
28											83.59%	86.73%									
29											81.67%	84.17%									
30											80.24%	83.69%									
31											64.58%	76.67%									

**Table A3:** Definition of the minimum number of training days required for each criterion within Filter 3. The highlighted cells show when the mean PICP of each criterion of Filter 3 improves the mean PICP of Filter 2.

Min Training Days		HTG_Work_±20			HTG_Work_±10			HTG_Work_±25%			HTG_Work_±20%			HTG_Work_±15%			HTG_Work_±10%			HTG_Work_±5%	
		+MAE+R2			+MAE+R2			+MAE+R2			+MAE+R2			+MAE+R2			+MAE+R2			+MAE+R2	
		Mean PICP			Mean PICP			Mean PICP			Mean PICP			Mean PICP			Mean PICP			Mean PICP	
		Filter 2	Filter 3		Filter 2	Filter 3		Filter 2	Filter 3		Filter 2	Filter 3		Filter 2	Filter 3		Filter 2	Filter 3		Filter 2	Filter 3
1		82.15%	71.27%		83.27%	65.48%		80.74%	67.08%		81.43%	73.40%		81.81%	78.69%		82.03%	77.71%		82.04%	78.17%
2		82.22%	72.37%		83.43%	66.85%		80.79%	68.19%		81.43%	73.40%		81.67%	78.58%		82.03%	77.71%		82.04%	78.17%
3		83.01%	78.55%		81.85%	72.96%		82.83%	73.63%		81.75%	77.86%		81.78%	81.62%		82.12%	80.66%		81.24%	81.93%
4		82.83%	78.48%		81.73%	75.90%		83.62%	74.97%		83.57%	78.66%		81.77%	81.21%		81.78%	81.50%		82.18%	83.21%
5		82.83%	77.83%		83.33%	83.33%		82.06%	73.53%		83.43%	78.27%		81.77%	81.21%		81.78%	81.50%		82.15%	83.54%
6		82.72%	79.06%					82.00%	71.25%		84.39%	80.72%		82.58%	82.81%		81.26%	80.98%		82.15%	83.54%
7		75.83%	76.20%					79.00%	71.67%		81.17%	79.92%		83.08%	85.54%		81.77%	80.48%		82.25%	83.45%
8		77.33%	78.17%					78.89%	73.61%		82.62%	83.81%		81.03%	82.95%		82.34%	80.73%		82.25%	83.45%
9		78.33%	87.50%								87.22%	91.67%		82.12%	84.39%		81.22%	79.58%		82.25%	83.45%
10																	81.85%	79.71%		82.25%	83.45%
11																	82.04%	80.21%		82.25%	83.45%
12																	83.27%	79.76%		81.86%	83.33%
13																	89.58%	84.03%		81.89%	83.71%
14																	93.06%	94.44%		81.33%	83.20%
15																	87.50%	87.50%		82.84%	84.11%
16																				84.04%	85.09%
17																				83.96%	85.49%
18																				83.08%	85.08%
19																				83.53%	85.88%
20																				83.53%	85.88%
21																				84.84%	86.35%
22																				84.84%	86.35%
23																				86.06%	86.89%
24																				86.06%	86.89%
25																				87.85%	88.54%
26																				83.33%	84.38%
